# Crystallization Behavior of CaO-SiO_2_-Al_2_O_3_-MgO-TiO_2_-FeO Slag with Different CaO/SiO_2_ Ratios

**DOI:** 10.3390/ma19081574

**Published:** 2026-04-14

**Authors:** Wu Zhu, Qianqian Ren, Shuang Cai, Junguo Li, Lanjie Li, Luyang Duan, Yanan Zeng, Yajun Wang, Bao Liu

**Affiliations:** 1 College of Metallurgy and Energy, North China University of Science and Technology, Tangshan 063210, China; zhuwu2025777@163.com (W.Z.); renqianqian1@126.com (Q.R.); caishuang@ncst.edu.cn (S.C.); ly04062025@163.com (L.D.); zengyanann@126.com (Y.Z.); wangyj@ncst.edu.cn (Y.W.); 2 Hebei Puyang Iron and Steel Co., Ltd., Handan 056307, China; 3 Institute of Materials Technology Research, HBIS Group Co., Ltd., Shijiazhuang 050023, China; 4 Yanzhao Iron and Steel Laboratory, North China University of Science and Technology, Tangshan 063210, China

**Keywords:** titanium-extracted tailing, crystallization behavior, phase evolution, microstructure

## Abstract

Titanium-extracted tailing is a by-product generated during titanium-bearing blast furnace slag treatment process. The crystallization behavior of the titanium-extracted tailing during the cooling process is significant to its utilization for glass ceramics preparation. In this work, the CaO-SiO_2_-Al_2_O_3_-MgO-TiO_2_-FeO slag was used to explore the effect of CaO/SiO_2_ ratios on titanium-extracted tailing crystallization. FactSage 8.2 calculation and mineralogical characterizations were conducted to investigate the phase and microstructure evolution during the slag cooling process. Single hot thermocouple technique (SHTT) was employed for in situ observation of the crystallization process of the slag during the cooling process. The obtained results indicated that the perovskite, melilite, spinel, diopside and anorthite phases would be crystallized during the cooling process when the CaO/SiO_2_ ratios of the slag were 0.7–1.1. Increasing the CaO/SiO_2_ ratio to 1.3 and 1.5 promoted the crystallization of olivine and merwinite phases, however, inhibited the crystallization of diopside and anorthite phases. The initial crystallization temperature and the liquid phase disappeared temperature of the slag enhanced with improving CaO/SiO_2_ ratios. The initial crystallization temperature was controlled by perovskite phase precipitation when the CaO/SiO_2_ ratios of slag reached 0.7–1.3. Whereas the initial crystallization temperature was controlled by the crystallization of spinel phase when the CaO/SiO_2_ ratio of slag was 1.5. The incubation time for crystal nucleation reduced with increasing CaO/SiO_2_ ratios that promoted slag crystallization. Moreover, increasing the CaO/SiO_2_ ratio from 0.7 to 1.5 enhanced the critical cooling rate from 4 °C s^−1^ to 11 °C s^−1^.

## 1. Introduction

Titanium-bearing blast furnace slag (TBBFS) is a by-product obtained during the vanadium–titanium magnetite smelting process in a blast furnace [1,2,3]. In vanadium–titanium magnetite rich regions, such as Panzhihua in China, iron is reduced and separated in metallic form during smelting, whereas titanium reacts with other mineral components and is enriched in slag, generating the TBBFS with TiO_2_ contents of approximately 18–25 wt.% [4,5,6,7]. Statistically, the annual output of TBBFS is increasing rapidly with a rate of approximately 3 million tons, with the cumulative amount exceeding 80 million tons in China [8]. Although a small portion of TBBFS has been recycled to produce glass ceramics and cementitious materials, its utilization rate remains low [9,10,11]. To address this issue, Panzhihua Iron and Steel Group Co., Ltd. developed a “high temperature carbonization-low temperature chlorination” process to treat TBBFS. This technology enables the recovery of more than 60% of titanium from TBBFS [12,13]. However, this process still generates a large amount of residue, referred to as titanium-extracted tailing. Currently, the titanium-extracted tailing is predominantly disposed of in landfills, which not only wastes a large amount of titanium-bearing resources but also poses potential risks to the environment. Therefore, developing an efficient and sustainable route for the utilization of titanium-extracted tailing is of great importance.

Titanium-bearing slags have been investigated as potential raw materials for glass ceramics production [10,11,14]. The prepared glass ceramics are widely applied as shielding materials, electrical materials and biological materials, etc. [15,16,17]. Using titanium-extracted tailing for glass ceramics preparation is a good approach for its high value-added utilization. Hui et al. [18] utilized titanium-extracted tailing, silica tailings, coal fly ash and SiC to produce ceramic foams via the one-step sintering method. The obtained results indicated that the addition of titanium-extracted tailing was conducive to bubble generation and homogeneous distribution. Under the conditions of titanium-extracted tailing:silica tailing:coal flay ash addition ratio of 3:6:1, sintering temperature of 1160 °C, foaming agent contents of 0.1–0.15 wt.%, ceramic foams with a thermal conductivity of 0.23–0.37 W mK^−1^, flexural strength of 3.2–4.9 MPa, bulk density of 0.74–0.96 g cm^−3^, and water absorption of 0.24–1.78% were produced. Zheng et al. [19] converted titanium-extracted tailing to high performance glass ceramics by adding waste glass through smelting followed by water-quenching and sintering processes. The addition of waste glass enhanced the SiO_2_ content of titanium-extracted tailing. After sintering the titanium-extracted tailing and waste glass mixture at 950 °C for 45 min, the prepared glass ceramics with the mineralogical compositions of (Mg,Fe,Al,Ti)(Ca,Mg,Fe)(Si,Al)_2_O_6_, CaMg(SiO_3_)_2_, and Mg_2_(SiO_3_)_2_ showed Vickers hardness of 21.3 GPa, linear shrinkage rate of 17.40%, water absorption rate of 0.060%, and bulk density of 2.75 g cm^−3^. Chen et al. [20] prepared diopside-like based ceramics using coal fly ash and titanium-extracted tailing. Adding coal fly ash supplemented Al and Si of titanium-extracted tailing. By doping 20% coal fly ash into titanium-extracted tailing, the melilite would be transformed to diopside at 800–1100 °C and become stable at 1160–1200 °C. After sintering at 1180 °C for 60 min, diopside-like based ceramics with acid resistance of 97.36%, water absorption of 1.3%, bending strength of 141.8 MPa, bulk density of 2.89 g cm^−3^ and linear shrinkage of 13% were obtained.

The utilization of solid wastes for glass ceramics production mainly includes the high-temperature melting method [21,22], sintering method [23,24,25], and petrurgic method [26,27]. Compared to the other two methods, the petrurgic method regulates the crystal nucleation and growth by controlling the cooling process of the molten solid waste, avoiding the reheat treatment step, which is an energy saving and economical route. However, the petrurgic method also faces several limitations, including challenges in controlling type, nucleation and growth of the crystal during the cooling process. Titanium-extracted tailing mainly consists of CaO (30–35 wt.%), SiO_2_ (21–27 wt.%), Al_2_O_3_ (8–18 wt.%), TiO_2_ (9–14 wt.%), MgO (7–9 wt.%), and iron oxides (2–6 wt.%) [28,29,30]. The CaO and SiO_2_ contents account for more than 50 wt.% of the total amount of titanium-extracted tailing. The CaO/SiO_2_ ratio would significantly influence the crystallization of titanium-extracted tailing during the cooling process. Wu et al. [31] reported that the initial crystallization temperature of argon oxygen decarburization slag reduced with a decreasing CaO/SiO_2_ ratio. With the reduction in the CaO/SiO_2_ ratio, the dicalcium silicate, merwinite, and bredigite phases would transform to an amorphous state during the cooling process. Liu et al. [32] also reported that enhancing the CaO/SiO_2_ ratio increased the initial crystallization temperature of blast furnace slag. Wang et al. [33] investigated the crystallization behavior of the CaO-SiO_2_-MgO-Al_2_O_3_ slag. The results of continuous cooling transformation (CCT) and time-temperature-transformation (TTT) curves showed that the initial crystallization temperature decreased and the incubation time for crystallization increased with decreasing SiO_2_ content of slag. The morphology, content, type and size of the crystallized minerals were significantly influenced by CaO/SiO_2_ ratios. Currently, the research on crystallization behavior of titanium-extracted tailing during the cooling process is insufficient. The related work is urgently required for the efficient utilization of titanium-extracted tailing.

In this work, the CaO-SiO_2_-Al_2_O_3_-MgO-TiO_2_-FeO slag was used to explore the effect of CaO/SiO_2_ ratios on titanium-extracted tailing crystallization during the cooling process. The prediction of crystallization type, amount and temperature during the cooling process was conducted by FactSage 8.2 calculation. X-ray diffraction (XRD), high-resolution transmission electron microscopy (HRTEM), and field-emission scanning electron microscopy (FESEM) characterizations were carried out to explore the phase and morphology evolution during the cooling process. The slag crystallization process was observed in situ by applying a single hot thermocouple technique (SHTT). Based on the SHTT analysis, the TTT and CCT curves were constructed.

## 2. Materials and Methods

### 2.1. Raw Materials

As mentioned above, the chemical compositions of titanium-extracted tailing are CaO (30–35 wt.%), SiO_2_ (21–27 wt.%), Al_2_O_3_ (8–18 wt.%), TiO_2_ (9–14 wt.%), MgO (7–9 wt.%), and FeO (2–6 wt.%), accounting for more than 90 wt.% of the total amount. To investigate the effect of CaO/SiO_2_ ratios on the crystallization of titanium-extracted tailing, the CaO-SiO_2_-Al_2_O_3_-MgO-TiO_2_-FeO slag with the chemical contents close to the titanium-extracted tailing was employed in this work. Analytical-grade chemical reagents were used to synthesize these slags. The contents of Al_2_O_3_, MgO, TiO_2_, and FeO in the slag were held constant at 14.00 wt.%, 9.00 wt.%, 10.00 wt.%, and 5.00 wt.%, respectively. While, the CaO/SiO_2_ ratio was variable, rising from 0.7 to 1.5. The detailed chemical component of the synthesized slag is shown in Table 1. The CaO (purity ≥ 99.0%) and Al_2_O_3_ (purity ≥ 99.0%) were purchased from Tianjin Shentai Chemical Reagent Co., Ltd. (Tianjin, China). TiO_2_ (purity ≥ 98.0%), SiO_2_ (purity ≥ 99.0%), and MgO (purity ≥ 98.0%) were supplied by Tianjin Komio Chemical Reagent Co., Ltd. (Tianjin, China). FeO (purity ≥ 99.9%) was obtained from Guangdong Wengjiang Chemical Reagent Co., Ltd. (Shaoguan, China). All of the analytical-grade chemical reagents used in this work are amorphous. The analytical-grade chemical reagents were dried, weighed, and mixed to prepare the CaO-SiO_2_-Al_2_O_3_-MgO-TiO_2_-FeO slags.

### 2.2. Sample Preparation

Each of the five samples was prepared individually with its own CaO/SiO_2_ ratio. The XQM-4 planetary ball mill (Xiangtan Xiangyi Instrument Co., Ltd., Xiangtan, China) was employed to mix the analytical-grade chemical reagents at 500 r min^−1^ for 4 h to ensure the homogeneous mixture of the slags. The mixed slag samples were sieved using a 200 mesh sieve and then dried using an oven at 105 °C for 8 h. After that, 50 g of the dried sample with particle size less than 74 μm was placed into a corundum crucible and melted using a resistant furnace. By using a heating rate of 5 °C s^−1^, the temperature was raised from 25 °C to 1500 °C and held at 1500 °C for 2 h to achieve complete melting of the slag. Afterwards, the molten slag was cooled to the target temperatures of 1100–1400 °C with a cooling rate of 3 °C s^−1^. The cooled slag was then held at the target temperature for 2 h. The slag sample was immediately water-quenched to terminate crystallization after the isothermal treatment. Both the melting and the cooling processes were performed under an air atmosphere at 1 atm. The water-quenched slag sample was dried at 105 °C for 8 h, followed by crushing and sieving. The slag powder with particle size smaller than 74 μm was used for mineralogical and morphological characterization.

### 2.3. FactSage Thermodynamic Calculation

FactSage thermodynamic calculations were performed to investigate the effect of the CaO/SiO_2_ ratio on the crystallization of CaO-SiO_2_-Al_2_O_3_-MgO-TiO_2_-FeO slag during the cooling process using FactSage version 8.2 software. The FactPS and FToxid databases were employed in this work. The atmospheric pressure was set as 1 atm. The temperature range was set from 1000 °C to 1500 °C with a temperature interval of 1 °C. By entering the slag compositions listed in Table 1 into the Equilib module, the evolution of phase types and mass fractions as a function of temperature were calculated. The crystallized phase types during the slag cooling process were determined based on the thermodynamic data of minerals in FactPS and FToxid databases.

### 2.4. Mineralogical and Morphology Characterization

Mineralogical compositions of the water-quenched slag samples were detected by XRD (Rigaku SmartLab SE, Tokyo, Japan). The XRD measurements were conducted at a 2θ range of 10–90° and a scanning rate of 5° min^−1^. The morphology of the cooled slag samples was observed by FESEM (ZEISS Sigma 360, Oberkochen, Germany). The transmission electron microscopy (TEM, JEOL JEM-F200, Tokyo, Japan) equipped with selected area electron diffraction (SAED) was used to identify the structure of the crystallized phases. The atomically resolved lattice fringes of the cooled slag were also characterized by HRTEM.

### 2.5. Single Hot Thermocouple Technique (SHTT)

To observe the crystallization process of the synthesized slag samples during the cooling process, SHTT was employed to conduct the in situ isothermal and continuous cooling tests. All the SHTT experiments were performed under an air atmosphere at 1 atm. The crystallization process was observed in real time through a video camera by SHTT. The real-time slag morphology was captured as an optical image at intervals of 1 s. The images obtained were analyzed using Image J version 2.3 software to determine the initial crystallization time and temperature. The crystallization processes of all the samples was observed and analyzed using this method, consistently. For the isothermal cooling tests, the slag sample was firstly heated from 25 °C to 1500 °C and then held at 1500 °C for 100 s to ensure complete melting. Subsequently, the molten slag sample was cooled to the target temperatures and then held isothermally. The interval between the target temperatures was set as 25 °C. Based on the obtained results of isothermal cooling experiments, the TTT curves of the slag samples during the cooling process were constructed. For the continuous cooling experiments, the slag sample was also heated from 25 °C to 1500 °C, and held at 1500 °C for 100 s. The molten slag sample was then cooled to 1000 °C at controlled cooling rates ranging from 1 °C s^−1^ to 12 °C s^−1^. The crystallization process under different cooling rates was monitored, and the corresponding CCT curves were established.

## 3. Results and Discussion

### 3.1. FactSage Analysis of Crystallization Behavior of the Synthesized Slag

**Figure 1:**
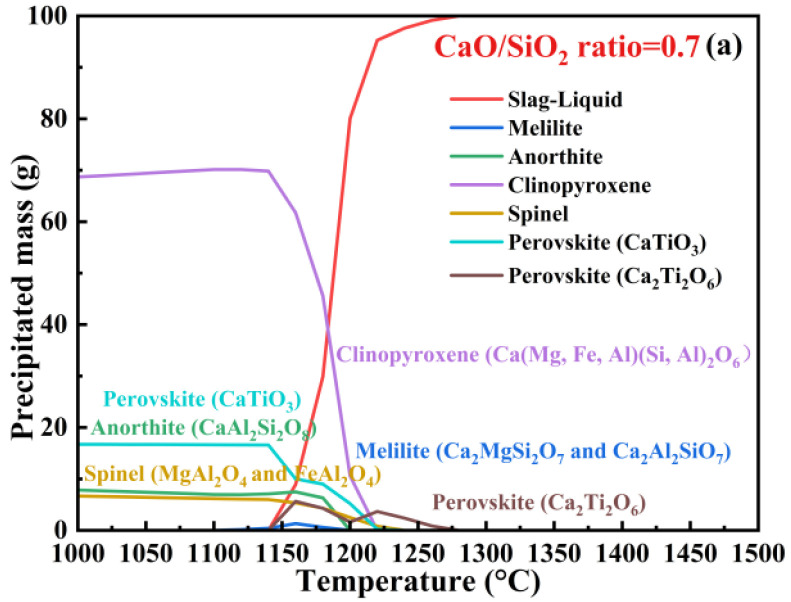

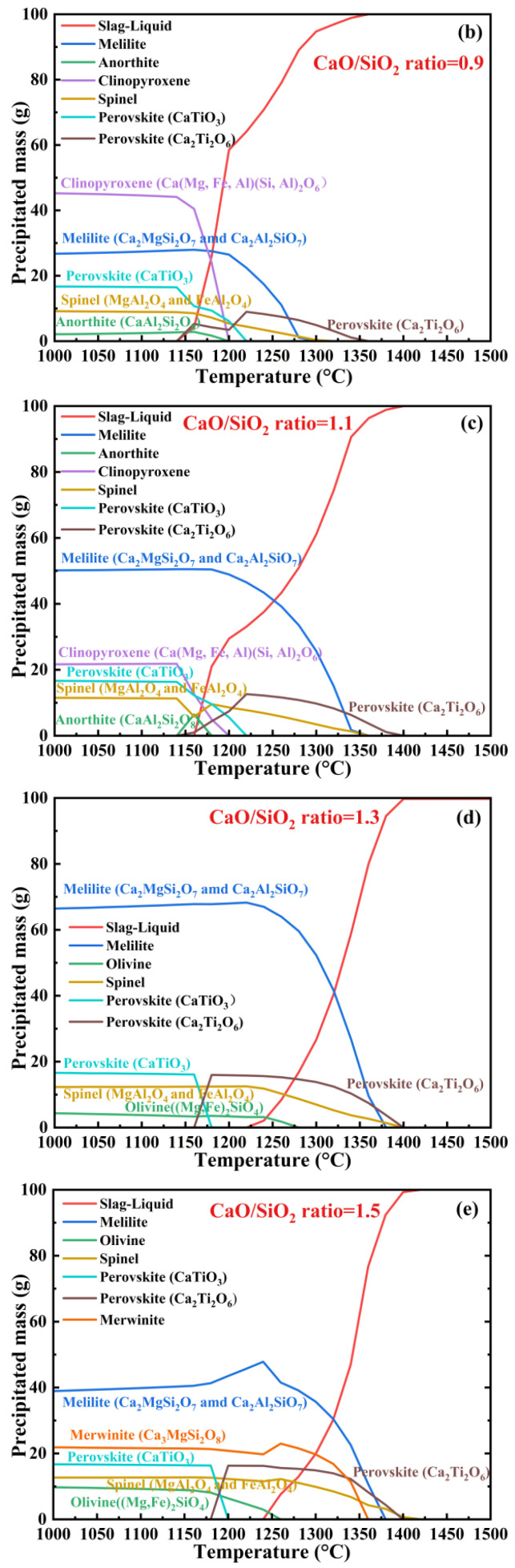
Thermodynamic equilibrium analysis of the crystallization behavior of the synthetized slag samples with different CaO/SiO_2_ ratios during the cooling process, (**a**) CaO/SiO_2_ ratio = 0.7, (**b**) CaO/SiO_2_ ratio = 0.9, (**c**) CaO/SiO_2_ ratio = 1.1, (**d**) CaO/SiO_2_ ratio = 1.3, (**e**) CaO/SiO_2_ ratio = 1.5.

Figure 1 exhibits the phase evolution of the synthesized slag samples with different CaO/SiO_2_ ratios during the cooling process. All of the slag samples existed as liquid state at 1500 °C. For the slag sample with a CaO/SiO_2_ ratio of 0.7 (Figure 1a), perovskite with the chemical formula of Ca_2_Ti_2_O_6_ was the first to crystallize when the temperature was reduced to 1270 °C, controlling the initial crystallization temperature. Subsequently, the spinel solid solution, which composed of MgAl_2_O_4_ and MgFe_2_O_4_, began to crystallize when this slag was cooled to 1236 °C. With the further decrease in temperature, perovskite (CaTiO_3_), clinopyroxene (Ca(Mg,Fe,Al)(Si,Al)_2_O_6_), anorthite (CaAl_2_Si_2_O_8_), and melilite solid solution (Ca_2_Al_2_SiO_7_ and Ca_2_MgSi_2_O_7_) would be precipitated in this order. The liquid phase disappeared temperature (LDT) of the slag with a CaO/SiO_2_ ratio of 0.7 was 1149 °C. As exhibited in Figure 1b,c, improving the CaO/SiO_2_ ratio from 0.7 to 1.1 did not change the crystallized phase type. The initial crystallization temperature enhanced from 1270 °C to 1389 °C and the LDT enhanced from 1149 °C to 1160 °C with the increase in CaO/SiO_2_ ratio from 0.7 to 1.1. As exhibited in Figure 1d, a new phase of olivine ((Mg,Fe)_2_SiO_4_) would be precipitated at 1271 °C; meanwhile, the clinopyroxene and anorthite phases were not crystallized during the cooling process when the CaO/SiO_2_ ratio reached 1.3. When the CaO/SiO_2_ ratio further improved to 1.5, another new phase of merwinite (Ca_3_MgSi_2_O_8_) was precipitated at 1358 °C (Figure 1e). It is noteworthy that the initial crystallization temperature improved to 1408 °C and was controlled by the crystallization of spinel phase when the CaO/SiO_2_ ratio reached 1.5.

Table 2 lists the initial crystallization temperatures of the mineralogical phases of the synthesized slag during the cooling process. In summary, improving the CaO/SiO_2_ ratio from 0.7 to 1.5 increased LDT from 1149 °C to 1257 °C and enhanced the initial crystallization temperature from 1270 °C to 1408 °C, respectively. This indicates that the improvement of the CaO/SiO_2_ ratio promoted slag crystallization. The initial crystallization temperature was controlled by the crystallization of the perovskite (Ca_2_Ti_2_O_6_) phase when the CaO/SiO_2_ ratios were 0.7–1.3, while it was controlled by the crystallization of the spinel phase when the CaO/SiO_2_ ratio was 1.5.

### 3.2. Mineralogical Composition and Microstructure of the Cooled Slag

**Figure 2:**
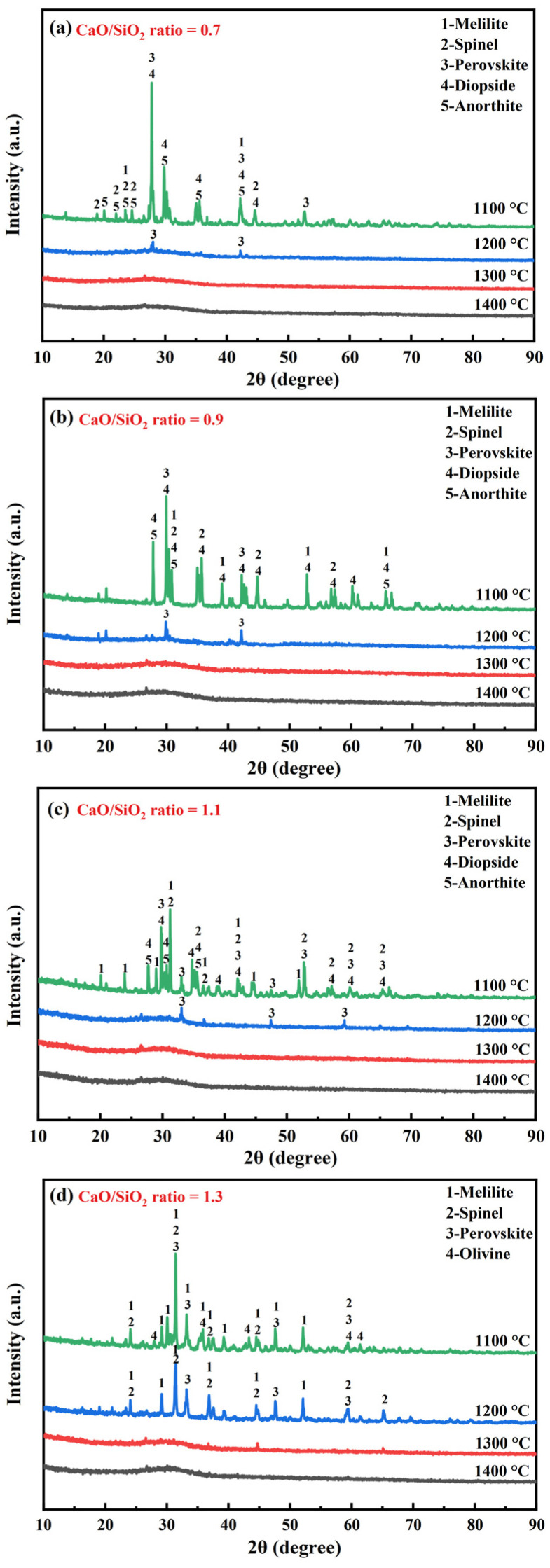

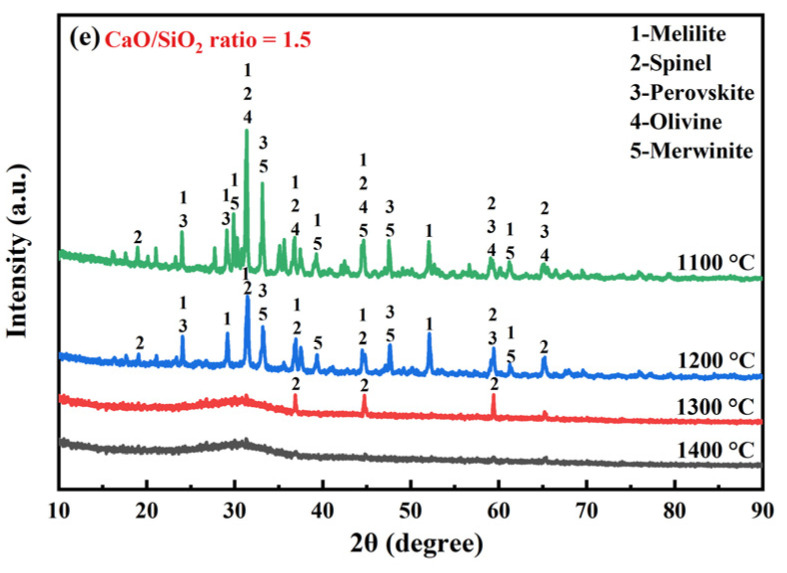
XRD patterns of the cooled slag samples with different CaO/SiO_2_ ratios, (**a**) CaO/SiO_2_ ratio = 0.7, (**b**) CaO/SiO_2_ ratio = 0.9, (**c**) CaO/SiO_2_ ratio = 1.1, (**d**) CaO/SiO_2_ ratio = 1.3, (**e**) CaO/SiO_2_ ratio = 1.5.

To validate the thermodynamic predictions and further elucidate the effect of the CaO/SiO_2_ ratio on the crystallization behavior of the synthesized slag, XRD measurements were performed. Figure 2 shows the XRD patterns of the water-quenched slag samples with different CaO/SiO_2_ ratios. As shown in Figure 2a–d, the XRD patterns of the slag with CaO/SiO_2_ ratios of 0.7–1.3 exhibited a broad amorphous halo when water-quenching at 1300 °C and 1400 °C. This indicates that these slag samples were predominantly glassy. For the slag with CaO/SiO_2_ ratios of 0.7–1.1, only the diffraction peaks corresponding to the perovskite phase were observed in the XRD patterns when water-quenching at 1200 °C. This suggests that the precipitation of the perovskite phase controlled the initial crystallization temperature of these slags. Improving the CaO/SiO_2_ ratio enhanced the intensity of diffraction peaks of the perovskite phase. This indicates that the crystallization of perovskite was promoted by enhancing the CaO/SiO_2_ ratio of the slag. For the slag with a CaO/SiO_2_ ratio of 1.3, besides the perovskite phase, the diffraction peaks corresponding to melilite and spinel were also observed in the XRD patterns when water-quenching at 1200 °C. While, for the slag with a CaO/SiO_2_ ratio of 1.5, the diffraction peaks corresponding to crystalline phases could be observed when this slag was water-quenched at 1300 °C (Figure 2e). The initial crystallized phase was spinel, which controlled the initial crystallization temperature. With the further decrease in temperature, the melilite, perovskite, olivine and merwinite phases would be crystallized.

All of the slag samples with high crystallinity can be obtained when water-quenching at 1100 °C. For the slag with CaO/SiO_2_ ratios of 0.7–1.1, the main crystallized phases were perovskite, melilite, spinel, diopside and anorthite. For the slag with a CaO/SiO_2_ ratio of 1.3, besides perovskite, melilite and spinel phases, a new phase of olivine was crystallized. Meanwhile, the crystallization of diopside and anorthite phases was inhibited. The further increase in the CaO/SiO_2_ ratio to 1.5 would crystallize another new phase of merwinite. It is noteworthy that the initial crystallization temperature obtained by XRD analysis was lower than that calculated by FactSage software. This can be attributed to the FactSage calculation being a thermodynamic analysis method without kinetics control. Due to the high viscosity of the molten slag and insufficient holding time, the experimental crystallization cannot reach the thermodynamic equilibrium, leading to the initial crystallization temperature obtained by the experiment being lower than the FactSage thermodynamic calculation [34].

**Figure 3:**
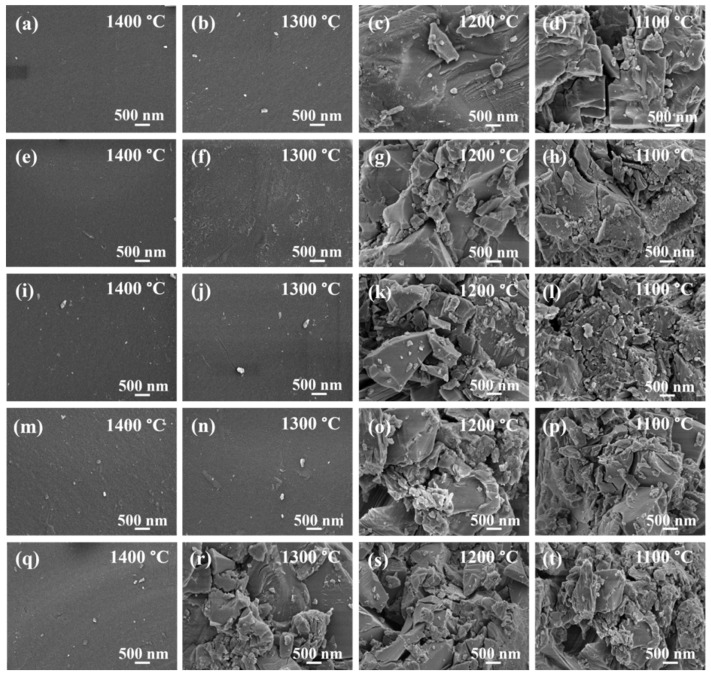
FESEM images of the cooled slag samples with different CaO/SiO_2_ ratios, (**a**–**d**) CaO/SiO_2_ ratio = 0.7, (**e**–**h**) CaO/SiO_2_ ratio = 0.9, (**i**–**l**) CaO/SiO_2_ ratio = 1.1, (**m**–**p**) CaO/SiO_2_ ratio = 1.3, (**q**–**t**) CaO/SiO_2_ ratio = 1.5.

To explore the influence of the CaO/SiO_2_ ratio on slag morphology, FESEM measurements were performed. Figure 3 exhibits the cooled slag morphology obtained by FESEM analysis. The slag samples with different CaO/SiO_2_ ratios exhibited smooth morphology when cooling at 1400 °C. This indicates that these slag samples were glassy. No crystalline phase was precipitated at 1400 °C, corresponding to the results of XRD analysis. With the decrease in temperature to 1300 °C, the morphology of the slag samples with CaO/SiO_2_ ratios of 0.7–1.3 remained smooth, while the rough morphology was observed from the slag with a CaO/SiO_2_ ratio of 1.5, indicating the crystallization of this slag at 1300 °C. With the further reduction in temperature below 1200 °C, the slag samples with CaO/SiO_2_ ratios of 0.9–1.5 obviously showed the rough morphology. It is noteworthy that the slag sample with a CaO/SiO_2_ ratio of 0.7 showed a small portion of roughly crystalline morphology when cooling at 1200 °C. However, most of slag particle areas still exhibited the smoothly amorphous morphology. This indicates that the crystallization amount of this slag was low when cooling at 1200 °C. The rough morphology can be obviously observed from the slag with a CaO/SiO_2_ ratio of 0.7 when cooling at 1100 °C.

**Figure 4:**
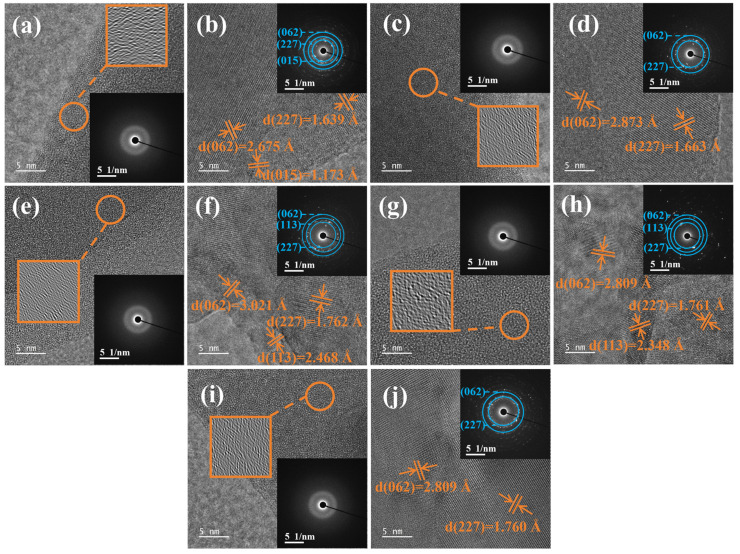
HRTEM images and corresponding SAED patterns of the cooled slag samples, (**a**) CaO/SiO_2_ = 0.7, 1200 °C, (**b**) CaO/SiO_2_ = 0.7, 1100 °C, (**c**) CaO/SiO_2_ = 0.9, 1300 °C, (**d**) CaO/SiO_2_ = 0.9, 1200 °C, (**e**) CaO/SiO_2_ = 1.1, 1300 °C, (**f**) CaO/SiO_2_ = 1.1, 1200 °C, (**g**) CaO/SiO_2_ = 1.3, 1300 °C, (**h**) CaO/SiO_2_ = 1.3, 1200 °C, (**i**) CaO/SiO_2_ = 1.5, 1400 °C, (**j**) CaO/SiO_2_ = 1.5, 1300 °C.

To further investigate the amorphous-to-crystalline transformation of the slag samples, the HRTEM and SAED characterizations were conducted. As shown in Figure 4a,c,e,g,i, irregular lattice fringes and amorphous diffraction halos were observed from the slag samples with a CaO/SiO_2_ ratio of 0.7 and cooled at 1200 °C, CaO/SiO_2_ ratios of 0.9–1.3 and cooled at 1300 °C, and a CaO/SiO_2_ ratio of 1.5 and cooled at 1400 °C. This indicates that the components in these slag samples mainly presented as amorphous state [35]. When cooling at 1100 °C, the lattice fringes of the slag with a CaO/SiO_2_ ratio of 0.7 became regular and the SAED pattern showed typical polycrystalline ring-like diffraction characteristic, indicating the high crystallinity of this slag (Figure 4b). The crystal interplanar spaces of 1.639 Å, 2.675 Å and 1.173 Å corresponded to the spinel (2, 2, 7) (ICDD: 01-071-3851), perovskite (0, 6, 2) (ICDD: 01-072-9970) and diopside (0, 1, 5) planes (ICDD: 01-072-1497), respectively. As for the slag samples with CaO/SiO_2_ ratios of 0.9 and 1.1 and cooled at 1200 °C (Figure 4d,f), besides the spinel (2, 2, 7) plane (ICDD: 01-074-9347 and 01-075-6826), the perovskite (0, 6, 2) plane (ICDD: 01-070-8503 and 01-089-0056) and the melilite (1, 1, 3) plane (ICDD: 01-073-2041) were also observed. The difference between the HRTEM and XRD analysis may be attributed to the crystallized amounts of perovskite and melilite being low. These two phases cannot be detected by XRD analysis. For the slag sample with a CaO/SiO_2_ ratio of 1.3 and cooled at 1200 °C (Figure 4h), the crystal interplanar spaces of 1.761 Å, 2.809 Å and 2.348 Å were observed from the HRTEM image, which corresponded to the spinel (2, 2, 7) (ICDD: 01-072-7246), perovskite (0, 6, 2) (ICDD: 01-070-7337) and melilite (1, 1, 3) planes (ICDD: 01-089-5917), respectively. The HRTEM results align well with the XRD analysis. While, for the slag sample with a CaO/SiO_2_ ratio of 1.5 and cooled at 1300 °C, the crystal interplanar spaces of 1.760 Å and 2.809 Å can be found from the HRTEM image, corresponding to the spinel (2, 2, 7) and perovskite (0, 6, 2) planes, respectively. This indicates that besides the crystallization of spinel, a small amount of perovskite can also be crystallized during the cooling process. It is noteworthy that the interplanar spaces of the crystallized phases were influenced by the CaO/SiO_2_ ratio. This may be attributed to the crystallized phases being mainly presented as solid solutions. The variety of the CaO/SiO_2_ ratio changed the chemical composition of these solid solutions, resulting in interplanar space variation [36].

### 3.3. Crystallization Behavior of the Slag Under Isothermal and Continuous Cooling Conditions

**Figure 5:**
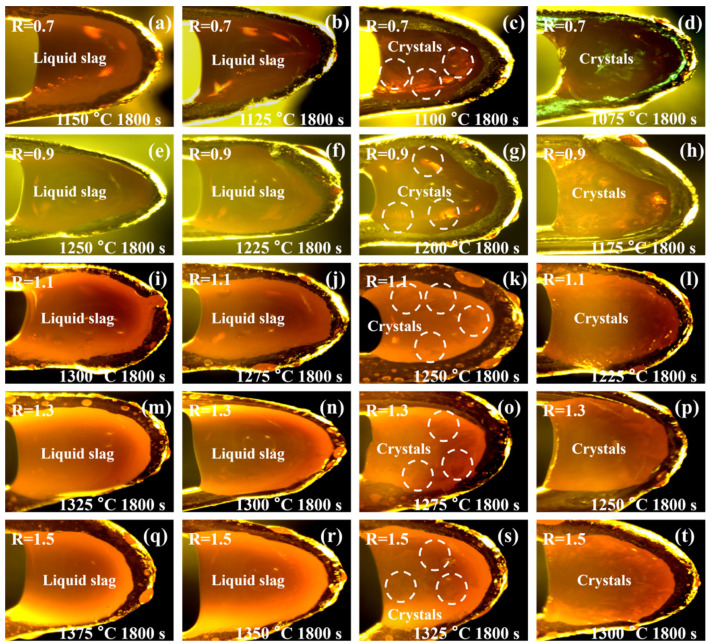
Crystallization images of the slag with different CaO/SiO_2_ ratios under isothermal cooling condition, (**a**–**d**) CaO/SiO_2_ ratio = 0.7, (**e**–**h**) CaO/SiO_2_ ratio = 0.9, (**i**–**l**) CaO/SiO_2_ ratio = 1.1, (**m**–**p**) CaO/SiO_2_ ratio = 1.3, (**q**–**t**) CaO/SiO_2_ ratio = 1.5.

Figure 5 exhibits the crystallization images of the slag samples under isothermal cooling. As shown in Figure 5a,b, the slag with a CaO/SiO_2_ ratio of 0.7 retained a stable melt when the temperature was higher than 1125 °C. No crystallization was observed during the isothermal cooling process. A small number of crystals with dendritic or angular morphology was observed when the temperature was decreased to 1100 °C (Figure 5c), indicating the start of the crystallization. The further reduction in temperature to 1075 °C significantly increased the crystal amounts of this slag (Figure 5d). With the enhancement of the CaO/SiO_2_ ratio from 0.7 to 1.5, the initial crystallization temperature increased from 1100 °C to 1325 °C (Figure 5g,k,o,s).

Increasing the CaO/SiO_2_ ratio promoted slag crystallization. This may be attributed to the increased CaO/SiO_2_ ratio, which promoted silicate, titanate, and aluminate network structure depolymerization, which reduced the energy barrier for crystallization [37]. The slag with a larger CaO/SiO_2_ ratio required a lower supercooling temperature to derive the rearrangement of atoms. In addition, enhancing the CaO/SiO_2_ ratio also reduced the slag viscosity, which promoted the diffusion of atoms and the [38]. The in situ observation also found that the molten slag started to crystallize from the edge to center. This may be attributed to the precipitation of crystals being dominated by heterogeneous nucleation during the slag cooling process. The edge of the U-shape hot thermocouple provided enough nuclei to ensure the heterogeneous nucleation of the crystals. The movement of the floating crystals was observed from the slag with a CaO/SiO_2_ ratio of 1.5 at the initial crystallization temperature and time. This is attributed to the low viscosity of this slag. With the further reduction in temperature and extension of time, the floating crystals were interlaced together. The crystal position was fixed, and the crystal size became larger.

**Figure 6:**
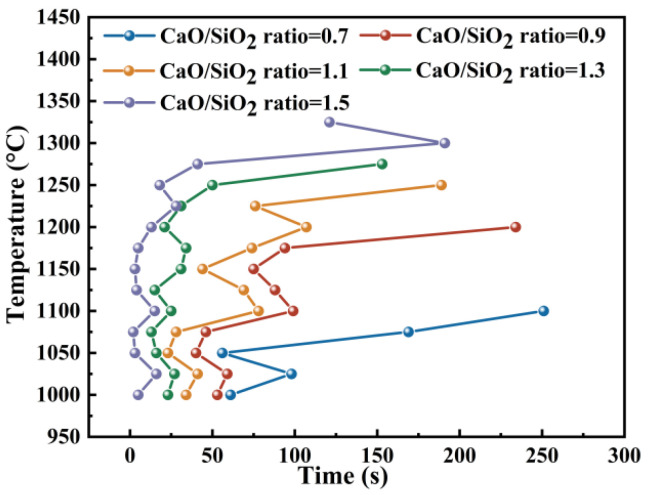
TTT curves of the slag with different CaO/SiO_2_ ratios under isothermal cooling condition.

Based on the initial crystallization temperature and time points collected from the isothermal cooling tests, the TTT curves were obtained. Figure 6 shows the TTT curves of the slag with different CaO/SiO_2_ ratios. The TTT curves showed typically double or triple “C” shapes, which is related to slag crystallization. This indicates that there are at least two or three kinds of phases that were crystallized during the isothermal cooling process [32]. The liquid phase would transform to solid phase when the free energy of the liquid phase is equal to or higher than the solid phase [34]. Increasing supercooling temperature can improve the driving force for this transformation. The “C” shape of the TTT curves is attributed to the evolution of the nucleation rate. The reduction in cooling temperature increased the supercooling degree of the molten slag that enhanced the driving force for nucleation and reduced the incubation time. The further decrease in temperature and the solid crystals precipitation increased the slag viscosity that inhibited the diffusion of the atoms and enhanced the incubation time. Therefore, the TTT curves showed a typical “C” shape.

The incubation time is the time required for initiating nucleation at a fixed supercooling temperature. The TTT analysis results showed that the incubation time decreased when improving the CaO/SiO_2_ ratio. This implies that the crystallization process was accelerated by enhancing the CaO/SiO_2_ ratio. In particular, the incubation time of the slag sample with a CaO/SiO_2_ ratio of 1.5 was near 0 s at the temperatures below 1250 °C, indicating a strong nucleation tendency. This may be attributed to the increasing CaO/SiO_2_ ratio reducing the viscosity of the molten slag that promoted the rearrangement of atoms for nucleation. It is noteworthy that the initial crystallization temperature obtained by TTT analysis was lower than the results predicted by FactSage calculation when reaching thermodynamic equilibrium. This is attributed to the supercooling degree being not sufficient for deriving crystallization at the calculated temperature. The crystallization of the slag needed quite a long time.

The initial crystallization temperatures obtained from FactSage calculation, quenched sample analysis and isothermal experiment are shown in Table 3. As shown in Table 3, the initial crystallization temperature calculated by FactSage was 100–200 °C higher than those obtained from the quenched sample analysis and isothermal experiment. This is attributed to the FactSage calculation being a thermodynamic analysis method without kinetics control. The FactSage calculation assumes that a sufficiently long time is provided for mineral crystallization. While, for the quenched sample and isothermal experiment, the holding times for mineral crystallization were 120 min and 30 min at the target temperature, respectively. The holding time may be insufficient. The crystallization of minerals requires a larger supercooling degree, resulting in the initial crystallization temperatures obtained from quenched sample analysis and isothermal experiment being lower than the FactSage calculation. For the slag with a CaO/SiO_2_ ratio of 0.7, the longer holding time led to the initial crystallization temperature obtained from the quenched sample analysis being 100 °C higher than that observed from isothermal experiment. While, for the slag with CaO/SiO_2_ ratios of 1.1–1.5, the difference between the quenched sample analysis and isothermal experiment can be attributed to the temperature interval set by the isothermal experiment being smaller. In actual production, the initial crystallization temperature obtained from the isothermal experiment may be the most reliable.

**Figure 7:**
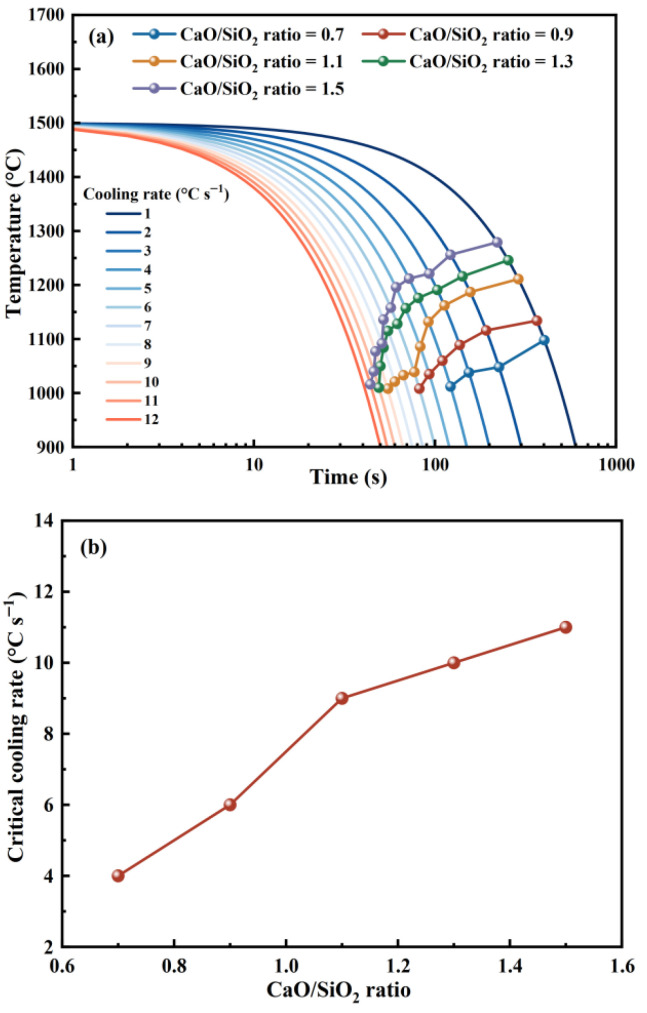
CCT curves and critical cooling rates of the slag with different CaO/SiO_2_ ratios under continuous cooling condition, (**a**) CCT curve, (**b**) critical cooling rate.

Continuous cooling tests were performed at controlled cooling rates from 1 °C s^−1^ to 12 °C s^−1^ to further explore the crystallization behavior. Based on the images collected from the continuous cooling tests, CCT curves were obtained. Figure 7 shows the CCT curves of the slag with different CaO/SiO_2_ ratios. As shown in Figure 7a, increasing the cooling rate decreased the initial crystallization temperature. This can be attributed to the high cooling rate not being able to provide enough time for the crystallization of the slag. The slag cooled at a high cooling rate requires a larger supercooling degree to drive its crystallization than the slag cooled at a low cooling rate [33]. Figure 7b shows that the critical cooling rate increased with increasing CaO/SiO_2_ ratios. The critical cooling rates of the slag with CaO/SiO_2_ ratios of 0.7, 0.9, 1.1, 1.3 and 1.5 were 4, 6, 9, 10, and 11 °C s^−1^, respectively. Crystallization occurred at a high cooling rate when the CaO/SiO_2_ ratio was high. This also indicates that increasing the CaO/SiO_2_ ratio can promote slag crystallization. This can be attributed to the increase in CaO/SiO_2_ ratio decreasing slag viscosity, which accelerated diffusion of atoms. Compared with the slag with a low CaO/SiO_2_ ratio, the crystallization of the slag with a high CaO/SiO_2_ ratio required a shorter time.

The CaO/SiO_2_ ratio is a key factor governing the crystallization behavior of the molten slag. As mentioned above, increasing the CaO/SiO_2_ ratio promoted crystallization and enhanced the critical cooling rate of the slag sample. The crystallization type and content were also influenced by the CaO/SiO_2_ ratio. As reported, the hardness, wear resistance and thermal stability can be improved by enhancing the crystalline phase amount of the glass ceramics [39]. However, excessive precipitation and growth of the crystalline phase would result in the reduction in densification degree and the enhancement of brittleness [40]. Controlling the glass and crystalline phase amounts is important for glass ceramics production. In addition, several studies indicated that the crystallization of the pyroxene phase can improve mechanical strength and hardness of glass ceramics [41]. The obtained results of this work indicate that the initial crystallization temperature and the critical cooling rate of the slag with a large CaO/SiO_2_ ratio are high, making the amorphous-to-crystalline transformation difficult to be precisely controlled. In addition, increasing the CaO/SiO_2_ ratio also inhibits the crystallization of the pyroxene phase. In view of this, the CaO/SiO_2_ ratios within the range of 0.7–0.9 are considered most suitable for glass ceramics production. Since the CaO/SiO_2_ ratio of titanium-extracted tailing is approximately 1.4, the modification of its chemical composition is required before being used for glass ceramics preparation.

Table 4 shows the investigations on the utilization of titanium-extracted tailing for glass ceramics preparation as reported in the literature and in this work. Currently, studies on the utilization of titanium-extracted tailing for glass ceramics preparation mainly focus on the optimization of sintering parameters, as well as the properties of the prepared glass ceramics. There are few reports on the utilization of titanium-extracted tailing for glass ceramics production using the petrurgic method. Research on crystallization behavior of titanium-extracted tailing during the cooling process is insufficient. This work investigated the effect of CaO/SiO_2_ ratios on the crystallization thermodynamics and kinetics of titanium-extracted tailing during the cooling process, which can lay a theoretical foundation for titanium-extracted tailing modification and crystallization control during the production process of glass ceramics using the petrurgic method.

## 4. Conclusions

In this work, the effect of CaO/SiO_2_ ratios on the crystallization behavior of the CaO-SiO_2_-Al_2_O_3_-MgO-TiO_2_-FeO slag was investigated. The perovskite, melilite, spinel, diopside and anorthite phases were crystallized during the cooling process when the CaO/SiO_2_ ratios were 0.7–1.1. Increasing the CaO/SiO_2_ ratio to 1.3 and 1.5 promoted the crystallization of olivine and merwinite phases, however, inhibited the crystallization of diopside and anorthite phases. The initial crystallization temperature and the liquid phase disappeared temperature of the slag enhanced when improving the CaO/SiO_2_ ratio. The initial crystallization temperature was controlled by perovskite phase precipitation when the CaO/SiO_2_ ratios of slag reached 0.7–1.3. Whereas the initial crystallization temperature was controlled by the crystallization of the spinel phase when the CaO/SiO_2_ ratio of slag was 1.5. Increasing the CaO/SiO_2_ ratio decreased the incubation time for crystal nucleation that promoted slag crystallization. In particular, the incubation time of the slag sample with a CaO/SiO_2_ ratio of 1.5 was near 0 s at temperatures below 1250 °C, which showed a strong crystallization tendency. In addition, increasing the CaO/SiO_2_ ratio from 0.7 to 1.5 enhanced the critical cooling rate from 4 °C s^−1^ to 11 °C s^−1^. In view of the control over the amorphous-to-crystalline transformation, a CaO/SiO_2_ ratio in the range of 0.7–0.9 is considered the most suitable for preparing glass ceramics using titanium-extracted tailing.

## Figures and Tables

**Table 1:** Chemical component of the synthesized slag samples (wt.%).

Slag	CaO/SiO_2_	CaO	SiO_2_	Al_2_O_3_	MgO	TiO_2_	FeO
R1	0.7	25.53	36.47	14.00	9.00	10.00	5.00
R2	0.9	29.37	32.63	14.00	9.00	10.00	5.00
R3	1.1	32.48	29.52	14.00	9.00	10.00	5.00
R4	1.3	35.04	26.69	14.00	9.00	10.00	5.00
R5	1.5	37.20	24.80	14.00	9.00	10.00	5.00

**Table 2:** Initial crystallization temperature of the mineralogical phases of the synthetized slag samples during the cooling process.

Slag Sample	R1	R2	R3	R4	R5
Perovskite (Ca_2_Ti_2_O_6_)	1270 °C	1350 °C	1389 °C	1400 °C	1398 °C
Spinel	1236 °C	1306 °C	1359 °C	1392 °C	1408 °C
Melilite	1189 °C	1286 °C	1342 °C	1371 °C	1377 °C
Perovskite (CaTiO_3_)	1220 °C	1220 °C	1213 °C	1179 °C	1200 °C
Clinopyroxene	1203 °C	1196 °C	1184 °C	-	-
Anorthite	1199 °C	1188 °C	1172 °C	-	-
Olivine	-	-	-	1271 °C	1259 °C
Merwinite	-	-	-	-	1358 °C
LDT	1149 °C	1155 °C	1160 °C	1230 °C	1257 °C
Initial crystallization temperature	1270 °C	1350 °C	1389 °C	1400 °C	1408 °C

**Table 3:** Comparison of the initial crystallization temperatures obtained from different methods.

CaO/SiO_2_ Ratio	0.7	0.9	1.1	1.3	1.5
FactSage calculation	1270 °C	1350 °C	1389 °C	1400 °C	1408 °C
Quenched sample analysis	1200 °C	1200 °C	1200 °C	1200 °C	1300 °C
Isothermal experiment	1100 °C	1200 °C	1250 °C	1275 °C	1325 °C

**Table 4:** Investigations on the utilization of titanium-extracted tailing for glass ceramics preparation as reported in the literature and in this work.

Purpose	Result	Refs.
Preparing ceramic foams by using titanium-extracted tailing.	By enhancing the SiO_2_ and Al_2_O_3_ contents of titanium-extracted tailing, glass ceramics with a flexural strength of 3.2–4.9 MPa can be prepared.	[18]
Investigating the formation mechanism of glass ceramics prepared using titanium-extracted tailing.	By enhancing the SiO_2_ content of titanium-extracted tailing, glass ceramics with Vickers hardness of 21.3 GPa can be prepared.	[19]
Preparing diopside-like based ceramics using titanium-extracted tailing.	By enhancing the SiO_2_ and Al_2_O_3_ contents of titanium-extracted tailing, glass ceramics with a bending strength of 141.8 MPa can be prepared.	[20]
Investigating the effect of CaO/SiO_2_ ratios on the crystallization behavior of titanium-extracted tailing.	Increasing the CaO/SiO_2_ ratio promotes crystallization. The CaO/SiO_2_ ratios of 0.7–0.9 are suitable for glass ceramics production.	This work

## Data Availability

The original contributions presented in the study are included in the article, further inquiries can be directed to the corresponding authors.
